# Day length is associated with physical activity and sedentary behavior among older women

**DOI:** 10.1038/s41598-018-25145-w

**Published:** 2018-04-26

**Authors:** Mitchell A. Schepps, Eric J. Shiroma, Masamitsu Kamada, Tamara B. Harris, I-Min Lee

**Affiliations:** 1 Laboratory of Epidemiology and Population Science, National Institute on Aging, Bethesda, MD USA; 2 Department of Social and Behavioral Sciences, Harvard T.H. Chan School of Public Health, Boston, MA USA; 3 Division of Preventive Medicine, Brigham and Women’s Hospital and Harvard Medical School, Boston, MA USA

## Abstract

Physical activity may be influenced by one’s physical environment, including day length and weather. Studies of physical activity, day length, and weather have primarily used self-reported activity, broad meteorological categorization, and limited geographic regions. We aim to examine the association of day length and physical activity in a large cohort of older women, covering a wide geographic range. Participants (N = 16,741; mean (SD) age = 72.0 (SD = 5.7) years) were drawn from the Women’s Health Study and lived throughout the United States. Physical activity was assessed by accelerometer (ActiGraph GT3X+) between 2011 and 2015. Day length and weather information were obtained by matching weather stations to the participants’ location using National Oceanic and Atmospheric Administration databases. Women who experienced day lengths greater than 14 hours had 5.5% more steps, 9.4% more moderate-to-vigorous physical activity, and 1.6% less sedentary behavior, compared to women who experienced day lengths less than 10 hours, after adjusting for age, accelerometer wear, temperature, and precipitation. Day length is associated with physical activity and sedentary behavior in older women, and needs to be considered in programs promoting physical activity as well as in the analyses of accelerometer data covering wide geographic regions.

## Introduction

Physical inactivity has been estimated to cause 5.3 million excess deaths annually, approximately the same as does smoking^1^. Improved understanding of the correlates and determinants of activity, including the meteorological environment, can help inform study design and analysis of physical activity data^2,3^.

The meteorological environment includes variables such as day length, temperature, and type and amount of precipitation. Previous studies have shown precipitation and extreme temperatures to be barriers to increased physical activity as well as an association between increased day length and activity levels,

however these studies have been limited by: (1) use of self-reported physical activity, (2) use of month or season as proxy for environmental factors, (3) not controlling for multiple meteorological factors concurrently, and (4) the study of small geographic regions^4–8^. Physical activity questionnaires typically summarize activity levels over a time period (e.g., month or year), and are subject to imprecise recall^9–11^. Meteorological factors vary during a given month or season and by region. Thus, examining day-to-day objective measures of both physical activity, and meteorological factors across a large region will allow more precise understanding of the relationships between day length, temperature, and precipitation with physical activity levels. Accelerometers provide objective, real-time measures of physical activity by detecting changes in motion. The few studies that have investigated the relation between meteorological variables and physical activity using objective measures like accelerometry have been conducted in small geographic regions and with small or medium sample sizes^12–19^. The Women’s Health Study, a longitudinal cohort of older women, provides one of the first opportunities to examine objectively measured physical activity in a large sample across a wide geographic area.

While temperature and precipitation can be ‘countered’ by performing physical activity indoors and/or wearing appropriate clothing, day length is immutable, short of changing one’s residence. However, day length can be accounted for in statistical analyses when sampling over a large geographic region as well as taken into consideration when designing physical activity intervention studies. Further, day length may be unique among meteorological variables because it influences circadian rhythm^20^. To provide robust, additional information on the association between physical activity and day length, we aimed to examine the association between day length and objective measures of physical activity from a large cohort of older women across a wide geographic region.

## Methods

### Study Population and Design

**Figure 1:**
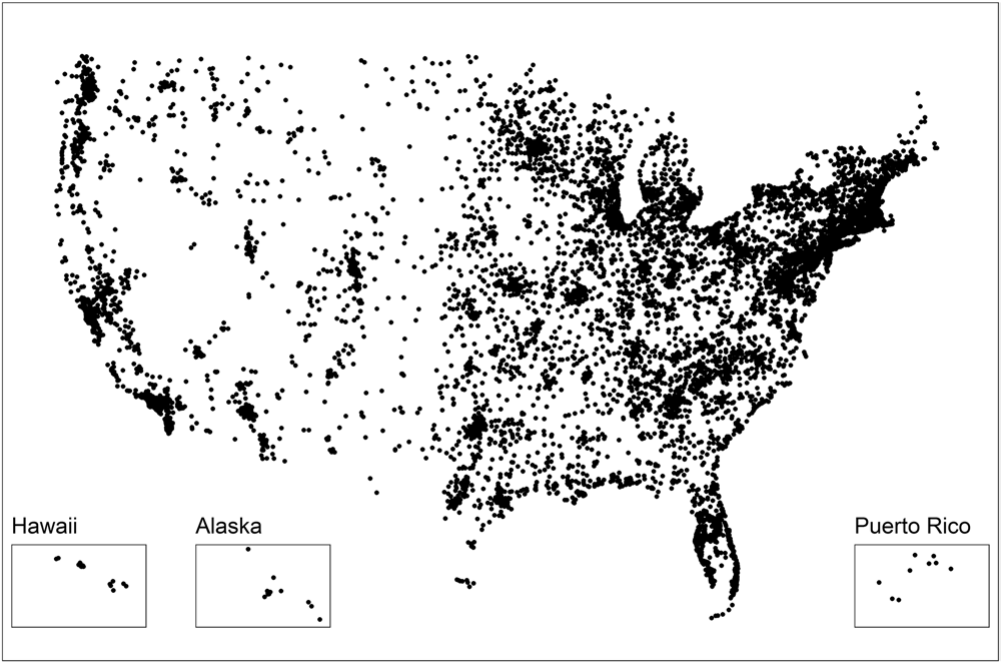
Map of participants, Women’s Health Study (2011–2015).

The Women’s Health Study is a completed randomized trial (1992–2004) studying the effect of vitamin E and aspirin on prevention of cardiovascular disease and cancer^21^. The trial included 39,876 healthy women (age ≥ 45 years) throughout the United States. Upon completion of the original trial in 2004, women were followed in an observational study. In 2011, an ancillary study was launched to examine accelerometer-assessed physical activity and mortality. Detailed methods are described elsewhere^22,23^. Briefly, 29,494 women were asked to participate in an ancillary study assessing physical activity levels using accelerometers. Of the 90% of invited women who responded, 1,456 women were ineligible due to being unable to walk outside the home unassisted and 6,931 women declined to participate. The remaining 18,289 women were mailed an accelerometer (ActiGraph GT3X+) on a belt and asked to wear the accelerometer on their hip during waking hours for 7 days. 17,466 women returned valid data (242 files were corrupt and 581 lost the monitors). Women who wore the accelerometer for at least 4 days for at least 10 hours each day were included in the analysis (n = 725 removed for insufficient wear time, described below)^24^. The final analysis sample was 16,741 older women living throughout the United States (Fig. 1). All methods were carried out in accordance with relevant guidelines/regulations. This research was approved by the institutional review board at the Brigham and Women’s Hospital. All participants provided written informed consent.

### Accelerometer-Assessed Physical Activity and Weather Variables

Accelerometer data were downloaded and aggregated to 60-second epochs. Accelerometer data were screened for wear using the participant wear-log diary and visual inspection^25^ combined with the Choi wear algorithm, a validated wear time algorithm based on tri-axial monitoring^26^. Briefly, on days the participant wore the monitor as determined by wear log or visual inspection, periods of non-wear were identified if no activity was detected for 90-minutes with a floating window of 30-minutes before and after the detection window. Valid wear days were defined as at least 10 hours of wear time per day. After identifying when the monitor was worn, we defined the physical activity intensity based on the number of accelerometer counts in all three dimensions (an aggregate of the acceleration over time), using validated cutpoints^27,28^. Moderate-to-vigorous intensity physical activity was defined as a minute where the accelerometer registered at least 2690 counts; light-intensity physical activity was defined as 200 to 2689 counts per minute; and sedentary activity as less than 200 counts per minute^27,28^. The number of steps per day were derived using the ActiGraph algorithm in Actilife (version 6, ActiGraph LLC, Pensacola, FL, USA).

Day length was defined as the difference between sunrise and sunset. Sunrise and sunset times were determined from the National Oceanic and Atmospheric Administration’s (NOAA) Solar Calculation algorithm (NOAA Solar Calculator, www.esrl.noaa.gov/gmd/grad/solcalc/). This algorithm uses a participant’s mailing address zip code and date in question to determine earth’s position relative to the sun. The latitude and longitude centroid of the participant’s mailing zip code were determined using CivicSpace US ZIP Code Database (CivicSpace Labs, Inc, 2004). Day length was obtained for each accelerometer wear day at the location of the mailing zip code. We then categorized day length into four categories, a priori, to ensure adequate distribution of participants: less than 10 hours, 10 to 12 hours, 12 to 14 hours, and greater than 14 hours.

Weather information was obtained from NOAA’s Global Historical Climatology Network for each participating weather station^29^. The participants’ latitudes and longitudes were used to identify the nearest weather stations that recorded data. For each participant and variable, the nearest station with records was used to retrieve data for each day of the wear period. The daily weather information extracted included maximum temperature, minimum temperature, rain (yes/no), snowfall (yes/no), and snow depth (yes/no). These latter three variables were combined to create a precipitation variable (yes/no).

### Statistical Analyses

Participant demographics and weather characteristics were described by each category of day length. The distribution of day length across accelerometer days was plotted using a histogram and boxplot for all participants. To visually inspect the relationship between day length and physical activity, median splines of physical activity measures (number of steps per day, minutes per day moderate-to-vigorous physical activity, light-intensity physical activity, and sedentary activity) were plotted against day length, as continuous variables.

To compare physical activity levels at varying day lengths, pairwise comparisons across day length categories were calculated by ANCOVA using the Tukey method to account for multiple comparisons. The ANCOVA adjusted for age, wear time, precipitation levels, temperature, snowfall, and snow depth. To estimate the mean physical activity level within each category, marginal mean values (95% confidence intervals) were estimated using least squares linear regression adjusting for age, wear time, precipitation levels, temperature, snowfall, and snow depth. We examined four physical activity measures: number of steps per day, and minutes per day of moderate-to-vigorous physical activity, light-intensity physical activity, and sedentary activity.

As discussed previously, regions may experience different meteorological characteristics, but the impact on physical activity within a given region is unclear^4,5,30^. Participants were classified by zip code into four regions; northeastern, southern, midwestern, and western, according to the United States Postal Service regional definitions. Day length distributions for each region was compared using boxplots. Differences in the association of day length and number of steps per day were examined by region. In addition, stratified analyses were conducted by age and body mass index. All stratified analyses adjusted for age, wear time, temperature, and precipitation.

Mixed linear models were used to analyze the data at the day-level, allowing for weather to change from day-to-day, and to account for the within-participant correlation from multiple days of data. Statistical analysis was performed in R (Version 3.4.3). Data reduction and processing were conducted using the high-performance computational capabilities of the Biowulf Linux cluster at the National Institutes of Health, Bethesda, MD (htttp://biowulf.nih.gov).

### Data Availability

Data are available from the Women’s Health Study Steering Committee upon reasonable request. Legal and ethical restrictions apply. Interested parties may email at whs@partners.org, and the following information will be required at the time of application: a description of how the data will be used, securely managed, and permanently deleted.

## Results

**Figure 2:**
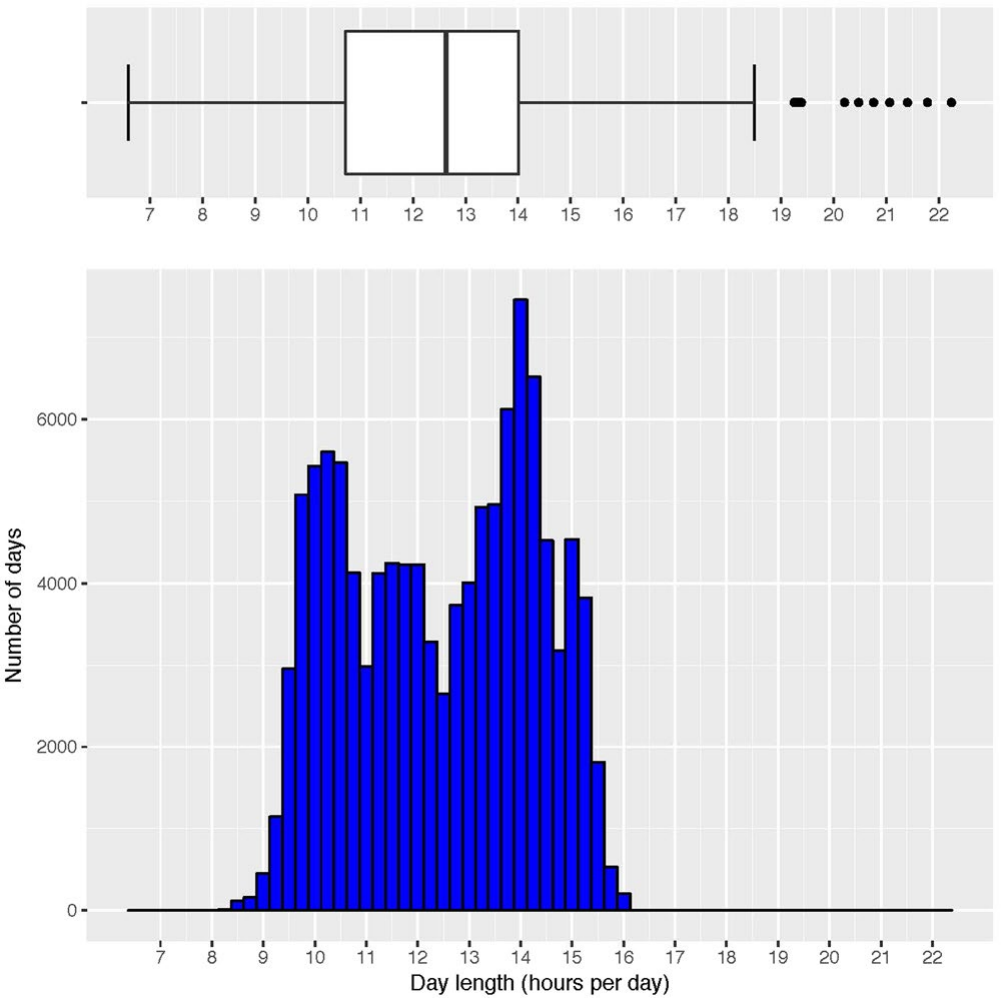
Histograph and boxplot of day lengths, Women’s Health Study (2011–2015).

On average, at the time of accelerometer wear, women were 72.0 (SD = 5.7, range = 62 to 101) years old and wore the accelerometer for 14.9 (SD = 1.7) hours per day. Participants experienced day lengths ranging from 6.6 to 22.2 hours with a mean day length of 12.5 (SD = 1.8) hours (Fig. 2). Of the 112,653 accelerometer days, 11.2% had day lengths of less than 10 hours, 31.6% had 10 to 12 hours, 31.9% had 12 to 14 hours, and 25.3% had greater than 14 hours of day length. Age (<10 hours: 71.1 years; 10–12 hours: 72.2 years; 12–14 hours: 72.1 years; 14+ hours: 71.8 years), wear time (<10 hours: 14.8 hours per day; 10–12 hours: 14.8 hours per day; 12–14 hours: 14.9 hours per day; 14+ hours: 15.0 hours per day), and BMI (<10 hours: 26.0 kg/m^2^; 10–12 hours: 26.3 kg/m^2^; 12–14 hours: 26.3 kg/m^2^; 14+ hours: 26.1 kg/m^2^) did not vary substantially across day length categories. The average distance of the weather stations used from the individual’s zip code was 3.2 (SD = 2.9) miles for rain data, 5.9 (SD = 4.1) miles for temperature data, 5.8 (SD = 14.8) miles for snowfall data, and 10.1 (SD = 9.9) miles for snow depth data.

**Figure 3:**
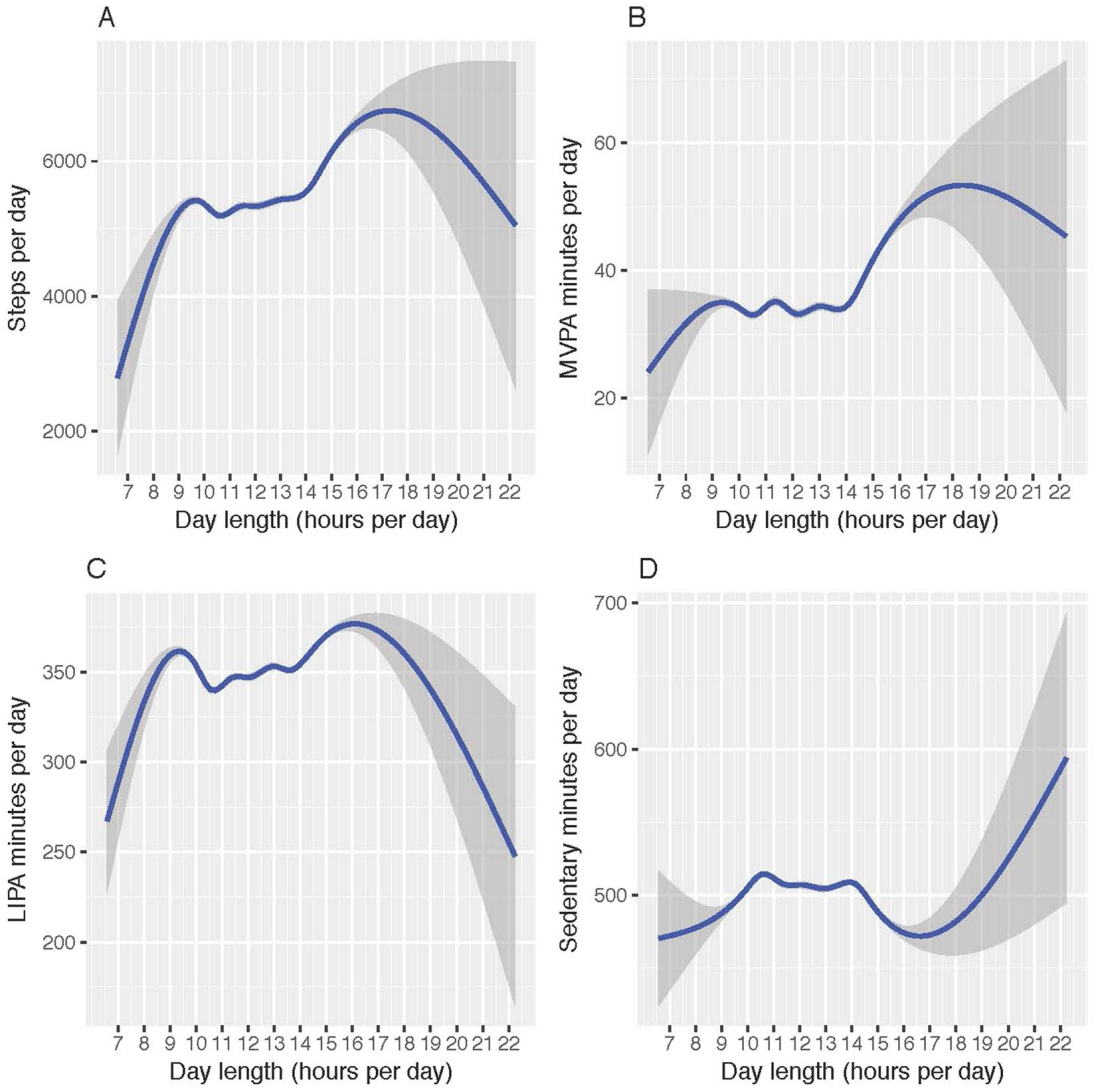
Median splines (95% confidence interval) of physical activity by day length, Women’s Health Study (2011–2015). (**A**) Steps per day. (**B**) Minutes of moderate-to-vigorous physical activity per day. (**C**) Minutes of light-intensity physical activity per day, (**D**) Minutes of sedentary activity per day.

To visually inspect the relationship between day length and physical activity, median splines with a 95% confidence interval of accelerometer measures were plotted by day length (Fig. 3). The number of steps per day and minutes per day of moderate-to-vigorous physical activity displayed similar relationships with day length; a non-linear dose response with the highest activity levels occurring around 17 and 18 hours of day length (Fig. 3A,B). Light-intensity physical activity displayed a similar trend to steps and moderate-to-vigorous physical activity, but with attenuated increase in activity between 9 and 18 hours of day length (Fig. 3C). Minutes of sedentary activity showed an inverse relationship to light-intensity physical activity, with the greatest sedentary time occurring during day lengths of 9 to 15 hours and days with greater than 19 hours of day length (Fig. 3D). For all activity estimates, confidence interval width was greatest at day lengths less than 9 hours and greater than 17 hours of day length due to fewer observed accelerometer days.

Pairwise comparisons and physical activity estimates (marginal mean (95% confidence interval)) were next calculated for each day length category (<10, 10–12, 12–14, and ≥14 hours) for number of steps per day and minutes per day of moderate-to-vigorous physical activity, light-intensity physical activity, and sedentary activity adjusting for age, accelerometer wear time, temperature, and precipitation (Table 1). The number of steps per day and minutes of moderate-to-vigorous physical activity from days with the longest day length (≥14 hours) were significantly greater compared to days with <14 hours of day length (p < 0.05). The minutes of light-intensity physical activity were fewer in days with 10 to 12 hours of day length compared to days with less than 10 hours, but were not significantly different in days with greater than 12 hours. Light-intensity activity was greater among days with ≥14 hours of day length compared to days with 12 to 14 or 10 to 12 hours. Sedentary behavior was lowest during days with ≥14 hours of day length compared to days with 12 to 14, 10 to 12, or <10 hours of day length. Sedentary behavior was also lower on days with <10 hours of day length compare to days with 10 to 12 hours, but not significantly different from days with 12 to 14 hours. Women experiencing day lengths ≥14 hours had 5.5% more steps, 9.4% more minutes of moderate-to-vigorous physical activity, and 1.6% less time spent in sedentary behavior compared to women experiencing day lengths <10 hours, after adjusting for age, wear time, temperature, and precipitation (Table 1).

**Table 1:** Physical activity by day length category, Women’s Health Study (2011–2015).

	Day length (hours per day)				Pairwise Differences*
	<10	10–12	12–14	≥14	
N accelerometer days	12 573	35 579	35 957	28 544	
Steps per day	5392.3 (5294.4, 5490.3)	5375.6 (5315.9, 5435.2)	5432.2 (5375.3, 5489.1)	5691.3 (5623.0, 5759.7)	C, E, F
MVPA minutes per day	34.1 (33.0, 35.3)	34.2 (33.5, 34.9)	34.3 (33.7, 35.0)	37.3 (36.5, 38.1)	C, E, F
LIPA minutes per day	355.4 (352.1, 358.7)	348 (345.9, 350.0)	351.8 (349.9, 353.7)	360.2 (357.9, 362.5)	A, D, E, F
Sedentary minutes per day	503.9 (500.2, 507.6)	510.7 508.4, 512.9)	506.9 (504.7, 509.0)	495.7 (493.1, 498.3)	A, C, E, F

Estimates are marginal means (95% confidence intervals) controlling for age, accelerometer wear time, temperature, and precipitation.

*Pairwise differences were computed with an alpha <0.05 after adjusting for multiple comparisons. Letters indicate a significant difference between two categories: A = <10 hours vs. 10–12 hours, B = <10 hours vs. 12–14 hours, C = <10 hours vs. ≥14 hours.

D = 10–12 hours vs. 12–14 hours, E = 10–12 hours vs. ≥14 hours, and F = 12–14 hours vs. ≥14 hours.

**Figure 4:**
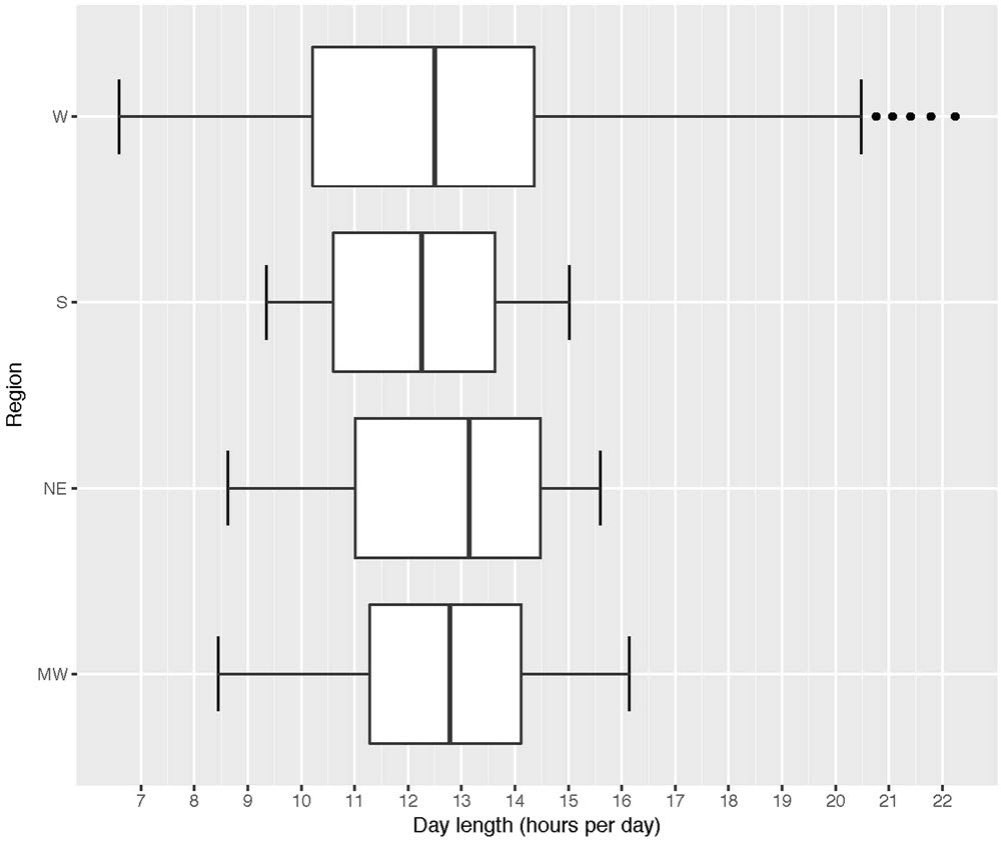
Boxplots of day length by United States region, Women’s Health Study (2011–2015). W = Western, S = Southern, NE = Northeastern, and MW = Midwestern.

To compare the relationship between physical activity (steps) and day length across regions in the United States, a stratified analysis was performed splitting the population into four regions (Northeast (NE), South (S), Midwest (MW), and West (W)). The range of day lengths differed by region (NE = 7.0, S = 5.7, MW = 7.7 and W = 7.7 hours per day), but there was substantial overlap of the day length distributions and similar median day lengths across all regions (Fig. 4). Regional differences in the association between day length and the number of steps were observed. The Midwest and Northeast regions showed the largest differences in steps between the longest and shortest days (13.3% and 8.5% increase respectively), but there was no significant differences in the number of steps across the day length categories for the West or South regions (Table 2). Similar to the pooled analysis, the longest days were associated with greater steps compared to the shortest days in the Northeast and Midwest regions (Table 2). A stratified analysis was also performed for age and body mass index. The longest days, compared to shortest days, had greater steps across age groups and among participants with a BMI of <25 and 25 to 30 (Table 2). Regional and stratified analyses adjusted for age, wear time, temperature, and precipitation (Table 2).

**Table 2:** Steps by day length categories according to region, age, and body mass index, Women’s Health Study (2011–2015).

	Day length (hours per day)				Pairwise Differences*
	<10	10 to 12	12 to 14	≥14	
**Region**					
South	5356.1	5277.1	5163.7	5167.6	—
	(5194.1, 5518.1)	(5164.3, 5362.7)	(5075.5, 5252.0)	(5000.7, 5334.4)	
Northeast	5463.6	5645.3	5820.5	5928.9	C, E
	(5211.1, 5716.2)	(5496.9, 5793.8)	(5679.9, 5961.0)	(5794.6, 6063.2)	
Midwest	4945.9	5262.8	5312.3	5605.7	B, C, E, F
	(4688.5, 5203.3)	(5157.6, 5368.1)	(5213.8, 5410.7)	(5489.1, 5722.4)	
West	5694.2	5579.3	5726.8	5805.1	—
	(5510.8, 5877.5)	(5434.6, 5723.9)	(5553.4, 5900.1)	(5658.9, 5951.4)	
**Age, years**					
<70	6220.9	6187.4	6186.0	6517.3	C, E, F
	(6050.8, 6390.9)	(6084.3, 6290.6)	(6086.8, 6285.1)	(6400.6, 6634.0)	
70–75	5384.7	5401.6	5508.8	5680.6	C, E
	(5207.2, 5562.2)	(5290.3, 5512.9)	(5406.2, 5611.4)	(5555.7, 5805.5)	
≥75	4256.6	4223.4	4258.5	4555.9	C, E, F
	(4101.2, 4412.1)	(4129.4, 4317.3)	(4166.8, 4350.1)	(4444.3, 4667.6)	
**Body Mass Index, kg/m** ^**2**^					
<25	6132.0	6059.4	6151.2	6403.0	C, E, F
	(5979.2, 6284.8)	(5963.9, 6154.9)	(6060.4, 6242.0)	(6295.6, 6510.3)	
25–30	5068.6	5210.1	5217.9	5511.6	C, E, F
	(4912.0, 5225.1)	(5117.2, 5303.0)	(5129.2, 5306.6)	(5404.4, 5618.8)	
≥30	4164.1	4165.0	4207.5	4378.8	E
	(3995.8, 4332.3)	(4066.0, 4263.9)	(4111.5, 4303.5)	(4262.6, 4495.0)	

Estimates are adjusted means (95% confidence intervals) controlling for age, accelerometer wear time, temperature, and precipitation.

*Pairwise differences were computed with an alpha <0.05 after adjusting for multiple comparisons. Letters indicate a significant difference between two categories: A = <10 hours vs. 10–12 hours, B = <10 hours vs. 12–14 hours, C = <10 hours vs. ≥14 hours.

D = 10–12 hours vs. 12–14 hours, E = 10–12 hours vs. ≥14 hours, and F = 12–14 hours vs. ≥14 hours.

## Discussion

This study shows a meaningful association between day length and objectively-measured physical activity in a large cohort of older women living throughout the United States. We observed that women experiencing the longest day length had with the greatest amount of physical activity, ranging from a 5 to 10% increase, independent of various weather conditions. These data expand upon previous research studies which also showed an association between day length and physical activity level, but were generally conducted using self-reported activity, using month or season to categorize differences in day length or weather, and limited to smaller geographic regions.

Both Chan *et al*. and Tucker *et al*. observed relationships between physical activity and day length, precipitation, temperature, and season in systematic reviews^4,5^. However, Tucker and Chan noted a need for objectively measured physical activity studies evaluating day-to-day variation in a variety of climatic zones^4,5^. Prior studies associating physical activity and climate have used telephone surveys or questionnaires to record physical activity levels in lieu of objective monitoring^8,31^. Accelerometers provide a more accurate assessment of physical activity, particularly across intensity levels. Few accelerometers adequately assess light-intensity physical activity. These studies have consistently indicated poor weather as a barrier to physical activity, however, these studies did not analyze specific days and types of weather^4^. Seasonal averages of weather aggregate data into specific epochs (weekly, monthly, or by season) and may lose the day to day variation in both activity and weather^9^. For example, if an individual’s monitoring week has rain every day, a seasonal or aggregated approach could fail to account for this anomaly.

Previous studies showed day length was a significant predictor of physical activity, however, these studies centered around one city analyzing one specific climate^7,12,18,32–34^. The physical activity levels of two older cohorts in Scotland were significantly associated with day length, analyzing regions of 20 and 30 mile radii^12,14^. Other studies incorporating participants in Kahoku, Japan (area = 25 mi^2^, n participants = 39 older adults), Perth, Australia (area = 2080 mi^2^, n participants = 1754 adults), and Prince Edward Island, Canada (area = 3517 mi^2^, n participants = 202 adults) each analyzed objective measures of physical activity, day length, and weather in one city and also found significant associations between rain, snow, temperature, duration of bright sunlight, and physical activity^7,18,34^. The Women’s Health Study reaches the wide range of the continental United States as well as Hawaii, Puerto Rico, and Alaska (area = 3,127,428 mi^2^).

We observed that the association of day length and physical activity differed by region of the United States. In the South and West, day length was not significantly associated with increased physical activity. This difference may be due to regional acculturation. For example, people living in southern states may be accustomed to the less-changing environment throughout the year and may not alter their habits as greatly compared to those in the northern states, with greater changes in day length and weather. Further investigation by region of habitant characteristics as well as other structural (parks, walking paths, public transit) and behavioral (such as cultural norms) factors may help explain the regional associational differences in activity level and day length.

The present study also presents some of the first examinations of the association of light-intensity physical activity and sedentary behavior with day length. Sedentary behavior was observed to be lower during the longest day length compared to the other day length categories. In addition, the shortest day length was also lower than 10 to 12 hour category, giving preliminary evidence to a U-shaped relationship. A similar, but opposite relationship, was observed for light-intensity activity. More research is necessary to examine the intensity of activity and its association with meterological factors.

Research examining day length and physical activity through the lens of circadian rhythm, in particular sleep patterns, is limited. However, our previous research has shown a co-dependence on sleep and physical activity^35^. The previous night’s sleep is associated with physical activity the next day, which in turns is associated with the later night’s sleep. For example, an association of less sleep was seen with subsequent lower activity, which was then associated with reduced sleep following the activity. This is an area of ongoing research interest.

While day length has been demonstrated to be associated with physical activity, it remains an immutable meteorological factor. However, day length may still be important in both the analysis of physical activity data as well as the design of physical activity studies. As studies of physical activity encompass larger geographic regions and longer durations, estimates of physical activity without adjusting for day length may not be comparable. This may be particularly important when estimating changes in activity levels within individuals over time. For example, comparing an estimate from a time with longer day lengths to shorter day lengths may be observed as decreased associated with an intervention, but could be an artifact of seasonality. One way to incorporate day length and weather into study design may be to do repeat sampling within similar times of year.

Recognition and implementation of knowledge of the association between the environment and physical activity have promoted physical activity through the development of indoor gymnasiums, covered paths and may increase physical activity levels in group activities^2,3,36–38^. If physical activity is less when the day is shorter, we can specifically promote interventions during these periods of the year at home, work or the community^38,39^. A more in-depth analysis examining during which hours of the day increases in physical activity during longer days occur is necessary to understand the interplay between occupational and recreational physical activity. Shifting occupational times, for example, could be piloted as a way to increase physical activity during the daylight.

A major strength of this study is that it is one of the first to examine a large sample of older women, over a large geographic area, using objective assessments capable of examining the day-to-day variations in both activity and day length. However, several limitations are worth mentioning. Participants are primarily older, white women and of higher socio-economic status, which may limit the generalizability of findings to other populations, including men and other age ranges. While we only collected one week of physical activity data, in a subset of women for whom two or three 1-week data collected over 2–3 years were collected, physical activity showed good reproducibility over this time frame^40^. This association study was a day level analysis. The daily weather data included variables collected throughout the entire day. If precipitation occurred during non-waking hours, it was included as a full precipitation day. Finally, we use women’s’ mailing zip codes to determine day length and weather. It is unknown how far women may have traveled while wearing the accelerometer. Newer devices with GPS may be used to further examine this issue.

In conclusion, extreme longer day lengths are associated with meaningful, higher levels of physical activity, even after adjusting for weather, among older women living throughout the United States. Day length should be taken into consideration when designing physical activity studies and analyses of accelerometer data across wide geographic regions.
